# Supplementary material for: Content-rich biological network constructed by mining PubMed abstracts
Source: BMC Bioinformatics. 2004 Oct 8;5:147. doi: 10.1186/1471-2105-5-147 (PMC528731; doi:10.1186/1471-2105-5-147)
Supplement: Additional File 2 — The original results of the above study (non-essential files are deleted to keep the file size under the limit set by BMC bioinformatics). [file 1471-2105-5-147-S2.bz2 › chilibotAdditionalFile2/dip10/590ID9195885E10962/html/NTHA_NTHB.html]

 


 **NTHA** and **NTHB** 
  
Found 122 abstracts in PubMed, retrieved 10.  
 

 What does Google say? 
 PDF only 
| .edu only 

---

**Interactive relationship** (e.g. stimulation, inhibition, etc)

**Non-interactive relationship** (e.g. studied together, co-existance, homology, etc.)

- ABN, AHN and AFN were converted by various microorganisms belonging to different bacterial genera e.g. Pseudomonas or Rhodococcus expressing either nitrilase  [ **NTHB** ]   [ **NTHA** ]  or nitrile hydratase activities.  Ref: 12845494 Appl Microbiol Biotechnol< MedlineTA>Appl Microbiol Biotechnol,
- Several plasmids were constructed to express the enzyme under the control of the lac promoter or its own promoter, together with or without nitrilase  [ **NTHB** ]   [ **NTHA** ]  and a possible regulatory protein that is present in the wild type genome.  Ref: 12651117 Protein Expr Purif, 2003
- Purification, cloning, sequencing and over expression in Escherichia coli of a regioselective aliphatic nitrilase  [ **NTHB** ]   [ **NTHA** ]  from Acidovorax facilis 72W.  Ref: 12655453 Appl Microbiol Biotechnol, 2003
- Site directed mutagenesis of the F. lateritium cyanide hydratase gene indicates that mutations leading to a loss of cyanide hydratase activity also lead to a loss of nitrilase  [ **NTHB** ]   [ **NTHA** ]  activity.  Ref: 12725921 FEMS Microbiol Lett, 2003
- The cyanide hydratase protein produced at a high level in Escherichia coli shows a low but significant nitrilase  [ **NTHB** ]   [ **NTHA** ]  activity with acetonitrile, propionitrile and benzonitrile.  Ref: 12725921 FEMS Microbiol Lett, 2003
- In order to suppress unwanted side reactions, biotransformations were performed with recombinant Escherichia coli strains that heterologously expressed nitrilase  [ **NTHB** ]   [ **NTHA** ]  activities originating from Pseudomonas, Rhodococcus, or Synechocystis strains.  Ref: 12845494 Appl Microbiol Biotechnol< MedlineTA>Appl Microbiol Biotechnol, 2003
- The cyanide hydratase enzyme of Fusarium lateritium also has nitrilase  [ **NTHB** ]   [ **NTHA** ]  activity.  Ref: 12725921 FEMS Microbiol Lett, 2003
- This suggests that the active site for cyanide hydratase and nitrilase  [ **NTHB** ]   [ **NTHA** ]  activity in the protein is the same.  Ref: 12725921 FEMS Microbiol Lett, 2003
- In addition, we report a rapid and convenient spectrophotometric method to monitor the activity of NI1 NHase  [ **NTHB** ]   [ **NTHA** ]  during purification.  Ref: 12729727 Protein Expr Purif, 2003
- A series of iron complexes based on the pentadentate ligand bis 2 methyl 2 mercaptopropyl 1 thia diazacyclononane, bmmp TASN 2, have been synthesized and characterized as models of iron containing nitrile hydratase NHase  [ **NTHB** ]   [ **NTHA** ] .  Ref: 12844310 Inorg Chem< MedlineTA>Inorg Chem, 2003
- Here we show yeast glutamine dependent NAD synthetase Qns1 requires both the nitrilase  [ **NTHB** ]   [ **NTHA** ]  related active site residues and the NAD synthetase active site residues for function in vivo.  Ref: 12771147 J Biol Chem< MedlineTA>J Biol Chem, 2003
- This is the first evidence of cyanide hydratase having nitrilase  [ **NTHB** ]   [ **NTHA** ]  activity.  Ref: 12725921 FEMS Microbiol Lett, 2003
- Earlier we discovered that glutamine dependent NAD synthetases contain N terminal domains that are members of the nitrilase  [ **NTHB** ]   [ **NTHA** ]  superfamily and hypothesized that these domains function as glutamine amidotransferases GATs for the associated synthetases.  Ref: 12771147 J Biol Chem< MedlineTA>J Biol Chem, 2003
- These data indicate that the nitrilase  [ **NTHB** ]   [ **NTHA** ]  related domain in Qns1 is the fourth independently evolved GAT domain to have been identified in nature.  Ref: 12771147 J Biol Chem< MedlineTA>J Biol Chem, 2003
- The mRNA expression of key enzymes involved in the synthesis, conjugate hydrolysis, accumulation and basipetal transport of auxin, namely indole 3 glycerol phosphate synthase, nitrilase  [ **NTHB** ]   [ **NTHA** ] , IAA amino acid hydrolase, chalcone synthase and PIN1 as an essential component of the basipetal IAA carrier, was investigated by reverse transcription polymerase chain reaction.  Ref: 12624772 Planta, 2003
- Earlier, we noted a perfect correlation between presence of a domain related to nitrilase  [ **NTHB** ]   [ **NTHA** ]  and glutamine dependence and then proved, in an accompanying paper, that the nitrilase  [ **NTHB** ]   [ **NTHA** ]  related domain is an essential, obligate intramolecular, thiol dependent glutamine amidotransferase in the yeast NAD synthetase, Qns1.  Ref: 12777395 J Biol Chem< MedlineTA>J Biol Chem, 2003
- The recombinant enzyme shows highest nitrilase  [ **NTHB** ]   [ **NTHA** ]  activity with benzonitrile.  Ref: 12725921 FEMS Microbiol Lett, 2003
- We report a novel heterologous system for the expression of catalytically active iron containing NI1 NHase  [ **NTHB** ]   [ **NTHA** ]  in Escherichia coli, involving coexpression with the E. coli GroES and GroEL chaperones.  Ref: 12729727 Protein Expr Purif, 2003
- A regioselective aliphatic nitrilase  [ **NTHB** ]   [ **NTHA** ]  from Acidovorax facilis 72W was purified and characterized, and the corresponding gene was cloned and sequenced.  Ref: 12655453 Appl Microbiol Biotechnol, 2003
- This nitrilase  [ **NTHB** ]   [ **NTHA** ]  gene was over expressed in Escherichia coli, generating a microorganism that efficiently and regioselectively catalyzes the conversion of aliphatic dinitriles to cyanocarboxylic acids.  Ref: 12655453 Appl Microbiol Biotechnol, 2003
- The nitrilase  [ **NTHB** ]   [ **NTHA** ]  activity is sufficient for growth of the recombinant strain on acetonitrile, propionitrile or benzonitrile as the sole source of nitrogen.  Ref: 12725921 FEMS Microbiol Lett, 2003
- Whereas AguA has no structural relationship to any known C N hydrolases, AguB is a protein of the nitrilase  [ **NTHB** ]   [ **NTHA** ]  family that performs thiol assisted catalysis.  Ref: 12634339 Microbiology, 2003
- Eukaryotic NAD synthetase Qns1 contains an essential, obligate intramolecular thiol glutamine amidotransferase domain related to nitrilase  [ **NTHB** ]   [ **NTHA** ] .  Ref: 12771147 J Biol Chem< MedlineTA>J Biol Chem, 2003
- The attempted conversion of the 2 acetoxynitriles to almost stoichiometric amounts of the corresponding 2 acetoxycarboxylic acids was finally achieved by using either a recombinant E. coli strain that highly overexpressed the nitrilase  [ **NTHB** ]   [ **NTHA** ]  gene from the pseudomonad or the purified enzyme derived from this strain.  Ref: 12845494 Appl Microbiol Biotechnol< MedlineTA>Appl Microbiol Biotechnol, 2003
